# Neuronal Autophagy: Self-eating or Self-cannibalism in Alzheimer’s Disease

**DOI:** 10.1007/s11064-013-1082-4

**Published:** 2013-06-05

**Authors:** Marzena Ułamek-Kozioł, Wanda Furmaga-Jabłońska, Sławomir Januszewski, Judyta Brzozowska, Małgorzata Ściślewska, Mirosław Jabłoński, Ryszard Pluta

**Affiliations:** 1 Laboratory of Ischemic and Neurodegenerative Brain Research, Mossakowski Medical Research Centre, Polish Academy of Sciences, Pawińskiego 5 Str, 02-106 Warsaw, Poland; 2 Department of Neonate and Infant Pathology, Medical University of Lublin, Chodzki 2 Str, 20-093 Lublin, Poland; 3 Department of Clinical Psychology, Medical University of Lublin, Chodzki 6, 20-093 Lublin, Poland; 4 Department of Orthopaedic and Rehabilitation, Medical University of Lublin, Jaczewskiego 8 Str, 20-954 Lublin, Poland

**Keywords:** Alzheimer’s disease, Autophagy, Amyloid precursor protein, β-Amyloid peptide, τ-Protein, Neuronal death

## Abstract

Autophagy is a major intracellular degeneration pathway involved in the elimination and recycling of damaged organelles and long-lived proteins by lysosomes. Many of the pathological factors, which trigger neurodegenerative diseases, can perturb the autophagy activity, which is associated with misfolded protein aggregates accumulation in these disorders. Alzheimer’s disease, the first neurodegenerative disorder between dementias, is characterized by two aggregating proteins, β-amyloid peptide (plaques) and τ-protein (tangles). In Alzheimer’s disease autophagosomes dynamically form along neurites within neuronal cells and in synapses but effective clearance of these structures needs retrograde transportation towards the neuronal soma where there is a major concentration of lysosomes. Maturation of autophago-lysosomes and their retrograde trafficking are perturbed in Alzheimer’s disease, which causes a massive concentration of autophagy elements along degenerating neurites. Transportation system is disturbed along defected microtubules in Alzheimer’s disease brains. τ-protein has been found to control the stability of microtubules, however, phosphorylation of τ-protein or an increase in the total level of τ-protein can cause dysfunction of neuronal cells microtubules. Current evidence has shown that autophagy is developing in Alzheimer’s disease brains because of ineffective degradation of autophagosomes, which hold amyloid precursor protein-rich organelles and secretases important for β-amyloid peptides generation from amyloid precursor. The combination of raised autophagy induction and abnormal clearance of β-amyloid peptide-generating autophagic vacuoles creates circumstances helpful for β-amyloid peptide aggregation and accumulation in Alzheimer’s disease. However, the key role of autophagy in Alzheimer’s disease development is still under consideration today. One point of view suggests that abnormal autophagy induction causes a concentration of autophagic vacuoles rich in amyloid precursor protein, β-amyloid peptide and the elements crucial for its formation, whereas other hypothesis points to marred autophagic clearance or even decrease in autophagic effectiveness playing a role in maturation of Alzheimer’s disease. In this review we present the recent evidence linking autophagy to Alzheimer’s disease and the role of autophagic regulation in the development of full-blown Alzheimer’s disease.

## Introduction

The word autophagy is derived from the Greek language auto (self) and phagy (eating) (self-eating) and generally refers to the cell catabolic and recycling processes in which cytoplasm non-essential and old or damaged components are transported to lysosomes for lysosome-mediated degradation and turnover process. It plays a significant homeostatic function in cells and keeps the balance between formation, synthesis, elimination and subsequent recycling of cell elements. De Duve [1] first used the word autophagy to describe an observable fact associated with single and/or double-membrane vesicles that had cytoplasm cargo inside at different stages of digestion/degradation. Autophagic cargo consists of cytoplasm constituents including misfolded or aggregated proteins, damaged organelles such as endoplasmic reticulum, mitochondria and peroxisomes as well as intracellular pathogens. Moreover, autophagy can be differentiated from heterophagy, in which the cell degrades extracellular substances. Autophagy machinery involves induction and presentation of target components such as organelles and proteins to the lysosomes via three major distinct autophagy processes: macroautophagy, microautophagy and chaperone mediated autophagy, which we will describe as autophagy which is the aim of this review. Autophagy is a degradation system/process involved in the clearance/elimination of aging/damaged organelles and pathologically changed proteins. During the process of autophagy, pre-autophagosomal structures, also called phagophores, fuse and elongate with engulfing the cytoplasm material within double-membrane vesicles, named autophagosomes. The autophagosomes initially fuse with endosomes to shape amphisomes, which later fuse with lysosomes where cytoplasm cargo is degraded. This machinery is implicated in various processes including e.g. cell differentiation and cell death [2]. Thus, a normal activity of autophagy guarantees the physiological turnover of damaged and aging organelles as a garbage recycling process. However, a considerable accumulation of autophagosomes is considered as an alternative route of cell death or an eventual cell survival pathway in response to a pathogen [3]. Autophagy today is recognized as an arbiter of neuron survival and death decisions in neurodegenerative disorders [4]. A characteristic element of many neurodegenerative diseases is the accumulation of intracellular protein aggregates. For example, in Alzheimer’s disease and after brain ischemia the presence of hyperphosphorylated tau protein and aggregated α-synuclein has been observed [5]. Given that the autophagy machinery is an important route by which intracellular proteins aggregates are cleared, it is of no surprise that autophagy is postulated to remove these neurotoxic aggregates [6]. It is evident that autophagy can play a role in cell death according to the type and degree of a pathogen [4]. Neuronal cell reaction might shift step by step from degradation of toxic proteins and damaged organelles via autophagy, which leads to its recovery and the initiation of apoptotic routes determining neuronal death. On the other hand, there is a suggestion that along with neurodegenerative diseases induction of autophagy is a protective reaction, and that the autophagy anomaly, rather than excessive autophagy, induces neuron death [2].

Autophagy in animals like in humans occurs physiologically and can be stimulated by starvation and different pathologies including Alzheimer’s disease. Besides its activity in maintaining cell homeostasis by producing substrates for both energy metabolism and vital protein synthesis as well as by supporting the clearance of damaged organelles and misfolded proteins, autophagy is important in various pathological episodes, including the degradation of aggregating proteins in neurodegenerative disorders. Dysfunction of the autophagy system has been presented to accompany a range of pathological situations including neurodegenerative disorders such as debilitating Alzheimer’s disease. In this review we will consider how autophagy is important in Alzheimer’s disease neuropathology.

## Amyloid Precursor Protein Processing by Autophagy

In a paper by Yu et al. [7] a novel β-amyloid peptide-generating pathway activated in Alzheimer’s disease has been presented, which occurs in autophagic vacuoles during their maturation defect in Alzheimer’s disease brain. It was shown that autophagic vacuoles significantly collect in Alzheimer’s disease brains. A great number of autophagic vacuoles were noted in dystrophic axons and neurites, earlier than the appearance of extracellular β-amyloid peptide as plaques in diseased brains indicating that autophagic vacuoles deposition/aggregation constitute an early hallmark of the disease in neurons. The above mentioned authors presented that β-amyloid peptide is generated within the autophagic vacuoles. To support their point of view they found β-amyloid peptides and γ-secretase on the membranes of autophagic vacuoles in Alzheimer’s disease brains. Additionally, in experiments in vitro they presented a correlation between autophagic activity and β-amyloid peptide production after starvation. On the other hand, β-amyloid peptide secretion was clearly suppressed when autophagy was inhibited e.g. by an excess of amino acids. The hypothesis that chronic and incomplete autophagic elimination involves itself in the overload formation of β-amyloid peptide today is officially approved of. The above correlate very well with the intracellular toxic properties of β-amyloid peptide [8]. Moreover, the new data presented by different laboratories fully support and provide clear insights into the role of autophagy in the processing of amyloid precursor protein and production β-amyloid peptide in Alzheimer’s brains [9–11].

## Autophagy and τ-Protein

Abnormal processing and altered clearance by autophagic-lysosomal machinery of τ-protein is implicated in the formation of neurofibrillary tangles in brains of Alzheimer’s disease [11]. The observations that have been presented are supported by a study of tau protein transgenic animals brains, which demonstrated altered autophagic-lysosomal system [12, 13]. Aforementioned models provoked the development of axonal spheroids, which were well-equipped with tau protein filaments and autophagosomes. Furthermore, this pathology appeared similar to abnormalities observed in the dystrophic neurites in Alzheimer’s disease brains [12, 13]. It was demonstrated that τ-protein aggregation and accumulation were induced not due to abnormalities in τ-protein phosphorylation. On the contrary, a changed activity of specific autophagic-lysosomal proteases was presented to be likely [14]. Other data showed that autophagy could eliminate soluble τ-protein, and its related aggregates and autophagy activity inhibition led to intensification in τ-protein aggregation and neurotoxicity [15]. Pathology of τ-protein in other models has also proved a link to autophagy abnormalities [11].

## Autophagy and Neuronal Death

Apart from adaption role of autophagy, it plays a role in cell death, as well [16]. Cell death is associated with continuous dysfunction of autophagy and may not be caused by the triggering of autophagy [17]. Other studies have demonstrated that autophagy might promote cell death [18, 19]. Autophagy-lysosome pathway dysfunction can cause neuron death [20] potentially associated with dysregulation of ubiquitin–proteasome pathway substrates [21]. A former and current study presenting ubiquitination as a significant regulatory element for autophagy [22–24], which is important as a key mechanism for clearing pathological protein aggregates from neuronal cells [25, 26], support the above mechanism. Regardless of the key role of above pathological proteins aggregates, β-amyloid peptide aggregates or neurofibrillary tangles their existence can be a sign of dysregulation or a problem of neuronal degradation mechanisms maturation [27]. It is becoming evident that autophagy can be a part of the cause in induction of cell death associating with different pathogens. Neuronal response might shift step by step from clearance of old proteins and damaged organelles via autophagy, which finally leads to its recovery to a normal condition, to the initiation of apoptotic mechanisms determining neuronal cell death. Finally, in the situation of neurodegenerative diseases the hypothesis is that generation of autophagy is a neuroprotective reaction and that defective or inadequate autophagy supports neuron death in the majority of these diseases, including Alzheimer’s disease [2, 20, 27, 28].

## Alzheimer’s Disease and Autophagy

Autophagosomes in Alzheimer’s disease brain are formed mainly in neurites and synapses. Active and efficient transport from synapses and neurites along microtubules to the neuronal body where lysosomes are accumulated is important for autophagic clearance in neuronal cells. In Alzheimer’s disease there occurs a massive concentration of autophagosomes in swelling and degenerating neurites due to problems with maturation of autophagosomes and their retrograde trafficking along defected microtubules towards the neuronal soma [10]. A common hypothesis is proposed that autophagy is developing in Alzheimer’s disease because of ineffective degradation of autophagosomes which hold amyloid precursor protein and secretases important for generation of β-amyloid peptides from amyloid precursor [7]. As a result β-amyloid peptides accumulate within autophagosomes in dystrophic neurites and become the most important intracellular pool of neurotoxic peptides in Alzheimer’s disease patients’ brains. It is documented that β-amyloid peptides can damage autophagosomes membranes and trigger the release of different toxic substances into cytoplasm compartment. It has been presented that accumulation of autophagosomes in neuronal cells can increase β-amyloid peptides plaques generation and that abnormal activity of autophagy especially in damaged neuropil could be an important source of toxic β-amyloid peptides [10, 11, 29]. In addition to this, the autophagy-related protein beclin 1 shows reduced expression in early Alzheimer’s disease, what induces β-amyloid peptides accumulation in transgenic mice overexpressing amyloid precursor protein due to reduced autophagy [30]. Thus, this observation is consistent with an observation that autophagosomes maturation is impaired in Alzheimer’s disease [30]. On the other hand, it is important to notice that β-amyloid peptides accumulation is affected by both generation and clearance [31–33]. Moreover, problems with autophagosomes maturation triggering β-amyloid peptides production, but decreased autophagy connected with beclin 1 deficiency, probably disturb β-amyloid peptides clearance by altered amyloid precursor protein metabolism [34].

## Alzheimer’s Disease Etiology and Amyloid

Despite ongoing interest in Alzheimer’s disease, the basis of this entity is not yet clear. At present, the best-established and accepted “culprit” in Alzheimer’s disease pathology by most scientists is the amyloid, as the main molecular factor responsible for neurodegeneration in this disease. Abnormal upregulation of amyloid production or a disturbed clearance mechanism may lead to pathological accumulation of amyloid in brain according to the “amyloid hypothesis” [31]. We and others critically reviewed these observations and highlighted inconsistencies between the predictions of the “amyloid hypothesis” and the published data [5, 32, 33]. There is still controversy over the role of amyloid in the pathological process. A question arises whether amyloid is responsible for the neurodegeneration or if it accumulates because of the neurodegeneration. Recent evidence suggests that the pathophysiology and neuropathology of Alzheimer’s disease comprises more than amyloid accumulation, τ-protein pathology and finally brain atrophy with dementia. Nowadays, a handful of researchers share a newly emerged view that the ischemic episodes of brain best describe the pathogenic cascade, which eventually leads to neuronal loss, especially in hippocampus, with amyloid accumulation, τ-protein pathology and irreversible dementia of Alzheimer type [5, 32, 33]. The most persuasive evidences come from investigations of ischemically damaged brains of patients and from experimental ischemic brain studies that mimic Alzheimer-type dementia [5]. The resulting brain ischemia dysregulated expression of amyloid precursor protein and amyloid-processing enzyme genes [32, 33] that, in addition, ultimately compromise brain functions, leading over time to the complex alterations that characterize advanced sporadic Alzheimer’s disease. Data suggest that ischemia promotes overproduction and aggregation of β-amyloid peptide in brain, which is toxic for ischemic neuronal cells as byproduct [5, 33]. The identification of the genes involved in Alzheimer’s disease induced by ischemia will enable to further define the events leading to sporadic Alzheimer’s disease-related abnormalities. Additionally, knowledge gained from the above investigations should facilitate the elaboration of the effective treatment and/or prevention of Alzheimer’s disease. Recently, increased autophagy has been reported in ischemic brain injury, too [35]. Autophagy can occur in neurons following brain ischemia giving mixed features of neuronal cell death [35]. The interaction after brain ischemia among autophagy, apoptosis and necrosis are complex with much crosstalk [35], and their roles in ischemia and Alzheimer’s disease need further studies.

## Conclusion

Aggregate-prone age-dependent and disease-related proteins like β-amyloid peptide and τ-protein can be a substrate of the autophagy activity. Still, the processes underlying the elimination/recycling of particular neurodegeneration-related proteins are now completely unclear. New data suggested possible influence of τ-protein dysfunction on autophagy clearance/elimination of cytoplasm garbage, which probably contributes to Alzheimer’s disease development/maturation [11]. τ-protein has been found to control the stability of microtubules but phosphorylation of τ-protein or an increase in the total level of τ-protein can cause dysfunction of neuronal cells microtubules. This put neurons in enormous danger due to abnormal function and placement of cellular organelles such as mitochondria and lysosomes [36]. In particular autophagosome degradation by lysosomes is a microtubule dependent route [37]. As a result, pathological activity of τ-protein may affect neuron function like neurites and axonal transport, which as well involves transportation of autophagic rubbish [38] and completely develops autophagosomes with all consequences in dystrophic axons and neurites in Alzheimer’s disease brains [29]. As a consequence of the above, autophagosomes dynamically form along neurites, axons and in synaptic area. However, effective clearance of these parts requires their retrograde transportation towards the neuronal body, where lysosomes are mainly packed. In Alzheimer’s disease brains the maturation of autophago-lysosomes in above parts of neurons and their retrograde transportation are obstructed, what leads to an enormous aggregation and accumulation of autophagy products (e.g. β-amyloid peptides) within dystrophic and degenerating axons and neurites. The combination of massive induced autophagy with faulty clearance of β-amyloid peptides producing autophagic vacuoles establishes circumstances helpful for β-amyloid peptides aggregation and accumulation in Alzheimer’s disease brains. Autophagy may contribute to Alzheimer’s disease amplification through secondary mechanisms such as dysregulated metabolism of amyloid precursor protein and increased production of β-amyloid peptides [5, 32, 33]. Autophagy is now recognized as an arbiter of neuronal survival and death decisions in Alzheimer’s disease. In conclusion, in full-blown Alzheimer’ disease, investigation of the regulation of autophagosome maturation and movement especially to neuronal cell body/soma is a promising area [24, 39]. Thus, autophagy regulation and stimulation might serve as a future therapy target for preventing and/or stopping Alzheimer’s disease neuropathogenesis.
